# Glacial refugia and speciation in a group of wind-pollinated and -dispersed, endemic Alpine species of *Helictotrichon* (Poaceae)

**DOI:** 10.1371/journal.pone.0205354

**Published:** 2018-10-15

**Authors:** Alexandra C. Ley, Jana Nissen, Alexandra Wölk, Martin Röser

**Affiliations:** Institute of Geobotany and Botanical Garden, University Halle-Wittenberg, Halle (Saale), Germany; University of North Carolina at Greensboro, UNITED STATES

## Abstract

In the Alps phylogeographic studies indicate for small insect-pollinated herbs that climatic fluctuations caused significant population migrations and fragmentations into glacial refugia at the periphery of the Alps. Here we investigate whether this holds also for wind-pollinated and -dispersed species. We therefore analysed the phylogeographic pattern (nuclear and chloroplast dataset) of a clade of the four species of the *Helictotrichon parlatorei* species group (Poaceae) endemic to the Alps. In contrast to earlier findings for small insect-pollinated herbs no clear barriers to gene flow could be detected in this species group. Instead a few haplotypes are widespread across the entire Alpine region. While the complete absence of a phylogeographic structure in the plastid dataset hints towards very efficient long distance seed dispersal, the moderate phylogeographic structure in the nuclear dataset indicates at least some spatial restriction to pollen dispersal. Rare haplotypes cluster solely in the Western and Southern central Alps and thereby suggest this to be the area of origin for the *H*. *parlatorei* species group from where expansion occurred following the presence of calcareous bedrock into the Eastern Alps. We thus conclude that the inclusion of taxa with complementary life-history traits is vital in understanding the glacial history of the Alpine flora.

## Introduction

The current distribution and intraspecific genetic structure of alpine species is thought to be mainly shaped by climatic conditions of the past. Here climatic fluctuations caused significant population migrations and fragmentations or even extinctions [1–3] and thereby repeatedly provoked allopatric speciation [4, 5].

**Fig 1:**
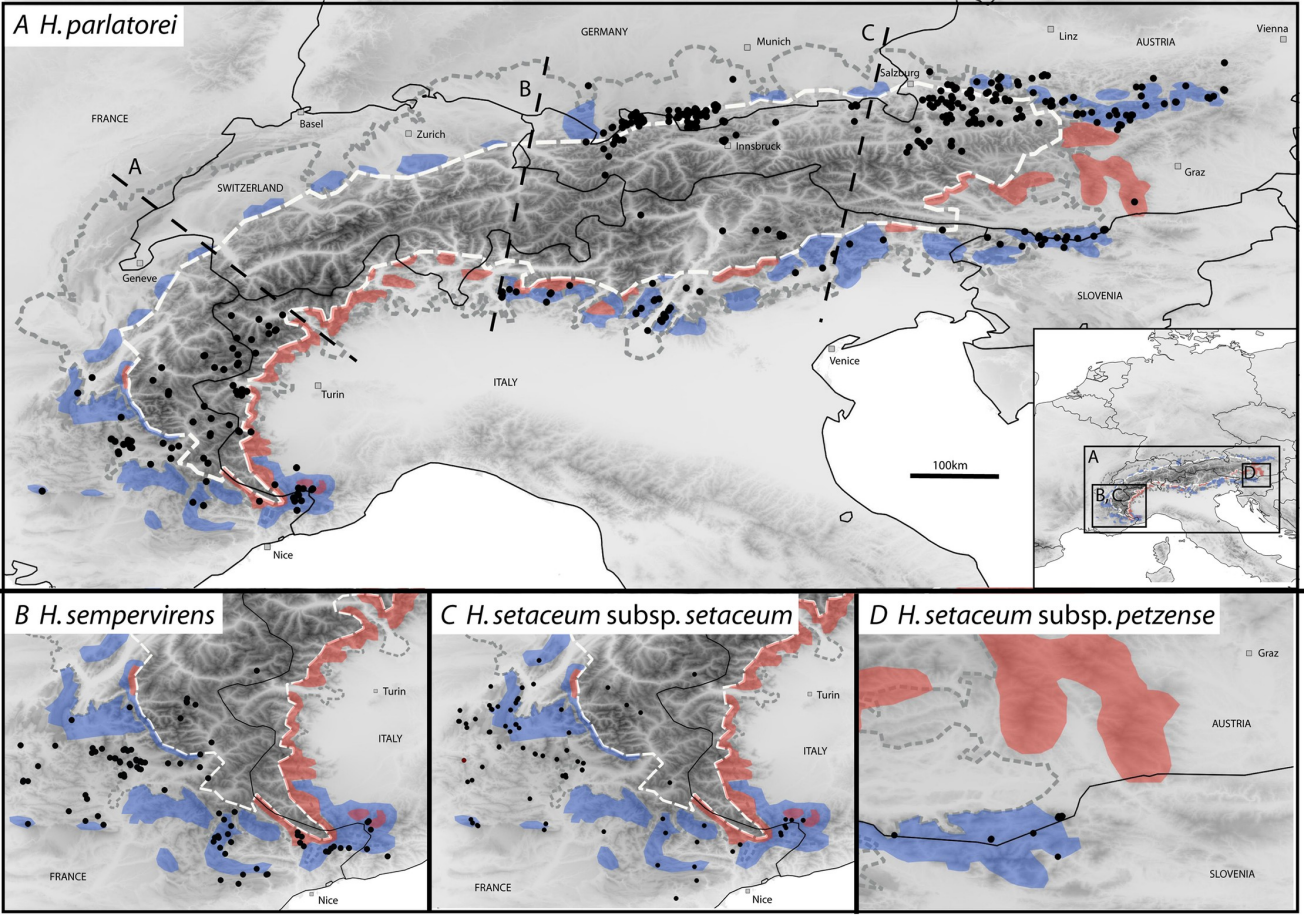
Distribution of the four taxa of the Alpine species group of *Helictotrichon parlatorei* species group (original maps). The maximum extent of the ice shield during the last glacial period is indicated (broken grey line; taken from [6] modified from [9], [10] and [11]). The snowline (i.e. altitude above which snow does not melt in climatically average years) during the last glacial maximum (LGM; 20.000 yr ago) is taken from [6] (after [12] and [13]). The snowline (broken white line) during the LGM was at about 1100 m a.s.l. in the northern and at about 2000 m a.s.l. in the central parts of the Eastern and Southwestern Alps. Broken black lines refer to three biogeographic regions (A–C; [6]). Glacial refugia on calcareous bedrock are in blue, on siliceous bedrock in red. Background: global SRTM version 4 digital elevation data [9].

A comparison of phylogeographic patterns with geological and palaeoenvironmental data demonstrates that glacial refugia were located during maximal glaciation along the southwestern, southern, eastern and northern border of the Alps [6]. Additional glacial refugia were present in central alpine areas, where plants from the alpine belt survived the last glaciation on ice-free mountain tops. The long-term glacial survival in isolated refugia in the periphery of the European Alps likely caused genetic drift, which explains the strong spatial genetic structure demonstrated in various molecular studies [6] and phenotypic differentiation consistent with adaptation to environmental conditions in glacial refugia [7]. The observed intraspecific phylogeographies suggest general patterns of glacial survival additionally tied to the adaptation to different bedrock: silicicolous versus calcicolous [8], which conform to well-known centres of alpine species diversity and endemism [6], i.e. common disjunctions at concordant biogeographic lines (Fig 1). However, such studies are based solely on small insect pollinated herbs with low dispersal abilities [5, 6]. Wind-pollinated and wind-dispersed species might be far less restricted to peripheral refugia due to a more effective dispersal mechanism. Their wide current distribution might not be based on ancient survival in refugia but effective long distance dispersal to adjacent and distant new habitats all across and around the Alps.

Here we are thus tackling the wind-pollinated and–dispersed perennial herbs of the grass genus *Helictotrichon* Besser (Poaceae), which is widely distributed in temperate Eurasia [14, 15]. The four alpine endemics belong to the *H*. *parlatorei* species group. They are characterized by an axis of the floret disarticulating only above the glumes (apomorphic character) and not below each floret as found in the remaining species of the genus [16]. Among the species of the *H*. *parlatorei* species group, *H*. *parlatorei* (J. Woods) Pilg. occupies the widest distribution extending from the Western to the Eastern Alps, however, with many gaps at the northern flank (Fig 1). It is also absent in the French Préalpes, the Swiss Alps and the central Austrian Alps [17]. Its distribution area is widely congruent with the regions of the Alps which remained unglaciated during the last glacial period [18, 19]. Further populations are found in the Italian and Austrian Central Alps. A few tetraploid populations are known from the Western Alps [17]. Two taxa, *H*. *sempervirens* (Vill.) Pilg. and *H*. *setaceum* (Vill.) Henrard subsp. *setaceum* are restricted to the Western Alps and French Préalpes (Fig 1). *Helictotrichon setaceum* subsp. *petzense* (H. Melzer) Röser (syn. *H*. *petzense* H. Melzer) is restricted to a few localities in the Southeastern Alps. The four taxa are distributed mainly in the montane to lower alpine belt up to 2600m a.s.l. with mostly southern exposition. All taxa are strongly basiphilic and usually grow on limestone or dolomite except for some populations of *H*. *parlatorei* in the Western Alps which are found on base-rich gneisses [16].

The aim of this study was to explain the present species and distribution pattern of the *H*. *parlatorei* species group endemic to the Alps: 1) Are the four alpine *Helictotrichon* taxa genetically well distinguished? 2) Do the phylogeographic patterns reflect the inter- and infraspecific geographic disjunctions between Western and Eastern Alps and Northern and Southern Alps? Are the centers of genetic diversity congruent with postulated refugia, and which migration scenarios can be extrapolated for each species? 3) What is the speciation scenario for the *H*. *parlatorei* species group?

## Material and methods

### Species description

The four alpine endemics of the *H*. *parlatorei* species group are characterized by specific morphological and anatomical features and a special mechanism of spikelet disintegration [14, 20–24]. Phylogenetic studies confirm the monophyly of the *H*. *parlatorei* species group ([22], but see [24]). The identity of its sister clade and the relationships among species within the *H*. *parlatorei* species group using genetic data remained however unresolved [22], although the four taxa are morphologically well distinguished [14, 21]. As with all other *Helictotrichon* groups, these species are also characterised by a considerable degree of polyploidy and some hybridisation [14, 22, 23, 25, 26].

Each species occupies a characteristic habitat: *H*. *parlatorei* (1500–2600 m a.s.l.) grows preferentially in montane to subalpine screes and dwarf-scrub communities [16]. In the Western Alps it can also be found in light subalpine *Larix* forests or even higher in thermophilic alpine *Sesleria* grassland. *Helictotrichon setaceum* subsp. *setaceum* (1000–2100 m a.s.l.) can be found in thermophilic plant communities on calcareous rock, occasionally, even on rocks in *Fagus* forests [16]. Quite similar habitats are occupied by subsp. *petzense* (1600–200 m a.s.l.), which is usually found on almost vertical and bare rocks [21, 27]. *Helictotrichon sempervirens* (600–2300 m a.s.l.) grows on thermophilic dry grassland but can also be found in open subalpine *Larix* and *Pinus sylvestris* forests and warm alpine *Sesleria* grassland [16].

### Plant material and taxon sampling

We sampled 133 individuals of *Helictotrichon* (98 *Helictotrichon parlatorei*, 11 *H*. *setaceum* subsp. *setaceum* and 10 subsp. *petzense*, 14 *H*. *sempervirens*) from herbarium and silica gel-dried material. Species identifications were thoroughly verified. Collection data, voucher information, and GenBank accession numbers are summarized in S1 & S2 Tables.

### DNA amplification

Total genomic DNA was extracted from leaf tissue using the NucleoSpin Plant-Kit (Macherey-Nagel, Düren, Germany) following the manufacturer’s instructions. Three plastid and one nuclear marker were amplified (Table 1). Amplification of the target loci was conducted in a Mastercycler (Eppendorf, Hamburg, Germany). Each 20 μl volume contains 2.00 μl 10× PCR buffer (without MgCl_2_), 1.00 μl DMSO, 0.20 μl 100 mM dNTPs, 1.4 μl 50 mM MgCl_2_, 0.20 μl 0.05 mM each forward and reverse primers, 0.20 μl *Taq* DNA polymerase (BioTherm DNA polymerase 5 u/μl from GeneCraft, Lüdinghausen, Germany), 1 to 2 μl genomic DNA extract and filled up with H2O. Amplification cycles were as follows: one cycle of 3 min at 94°C, 35 cycles of 30 s at 94°C, 30 s at 50°C, 1.5 min at 72°C with a final extension period of 10 min at 72°C. PCR products were sequenced by LGC Genomics (Berlin, Germany). Sequences were edited and then aligned manually (after preliminary automatic alignment in MUSCLE [28]) with the program Geneious 4.8.3 (http://www.geneious.com [29]) dx.doi.org/10.17504/protocols.io.taneide.[PROTOCOL DOI]

**Table 1:** Utilized primers, their sequence and references.

DNA-Region	Primer name	Sequence (5’ → 3’)	Reference
Plastid:			
*rps16*	*rps*F	GTG GTA GAA AGC AAC GTG CGA CTT	[51]
	*rps*R2	TCG GGA TCG AAC ATC AAT TGC AAT	[51]
*rpL*32-*trnL* (UAG)	*rpL*32-F	CAG TTC CAA AAA AAC GTA CTT C	[52]
	*trnL* (UAG)	CTG CTT CCT AAG AGC AGC GT	[52]
*ycf*3ln1	*ycf*3ln1-F	TGA CAG ATC ACG GCC ATA TT	[53]
	*ycf*3ln1-R	TTA YAG AGA TGG TGC GAT TT	[53]
*ycf*3ln2	*ycf*3ln2-F	GCY TGT TTC CAA TAC TCA GCA	[53]
	*ycf*3ln2-R	ATG GCC GTG ATC TGT CAT TA	[53]
Nuclear:			
*At103*	*At103*-F	CTT CAA GCC MAA GTT CAT CTT CTA	[54]
	*At103*-R	TTG GCA ATC ATT GAG GTA CAT NGT MAC ATA	[54]

### Phylogeographic analyses

For the nuclear and all combined plastid markers haplotypes were identified using DnaSP Version 5.10.1 [30]. Haplotype networks were calculated using Median Joining in Network 5.3 [31]. The geographic distribution of haplotypes was visualized in Q-GIS [32] summarizing individuals from nearby populations to a single population per area (S3 Table). The data were mapped onto the global SRTM version 4 digital elevation data [9]. The maximum extent of the Pleistocene ice shield during the last glacial period was taken from [6] (modified from [10–12]). The snowline (i.e. altitude above which snow does not melt in climatically average years) during the last glacial maximum (LGM; 20.000 yr bp) was also taken from [6] (after [13, 33]). The snowline during the LGM was at about 1100 m above sea level (a.s.l.) in the northern and at about 2000 m a.s.l. in the central parts of the Eastern and Southwestern Alps.

For widespread *Helictotrichon parlatorei* we also calculated a pairwise relatedness coefficient between individuals, Nij, accounting for the phylogenetic distance between alleles (‘‘ordered alleles”) using SPAGeDi Version 1.3. [34]. The level of population subdivision was assessed by the global G_ST_ (an estimator of F_ST_ where populations have equal weight, irrespective of sample size, [35]) as well as the corresponding parameter N_ST_ [35] which takes similarities between haplotypes into account, using SPAGeDi Version 1.3 [34]. As a minimum of three individuals per population is required for these statistics some populations had to be omitted from the analysis: Savoie and Tyrol (S3 Table).

## Results

### nDNA pattern

**Fig 2:**
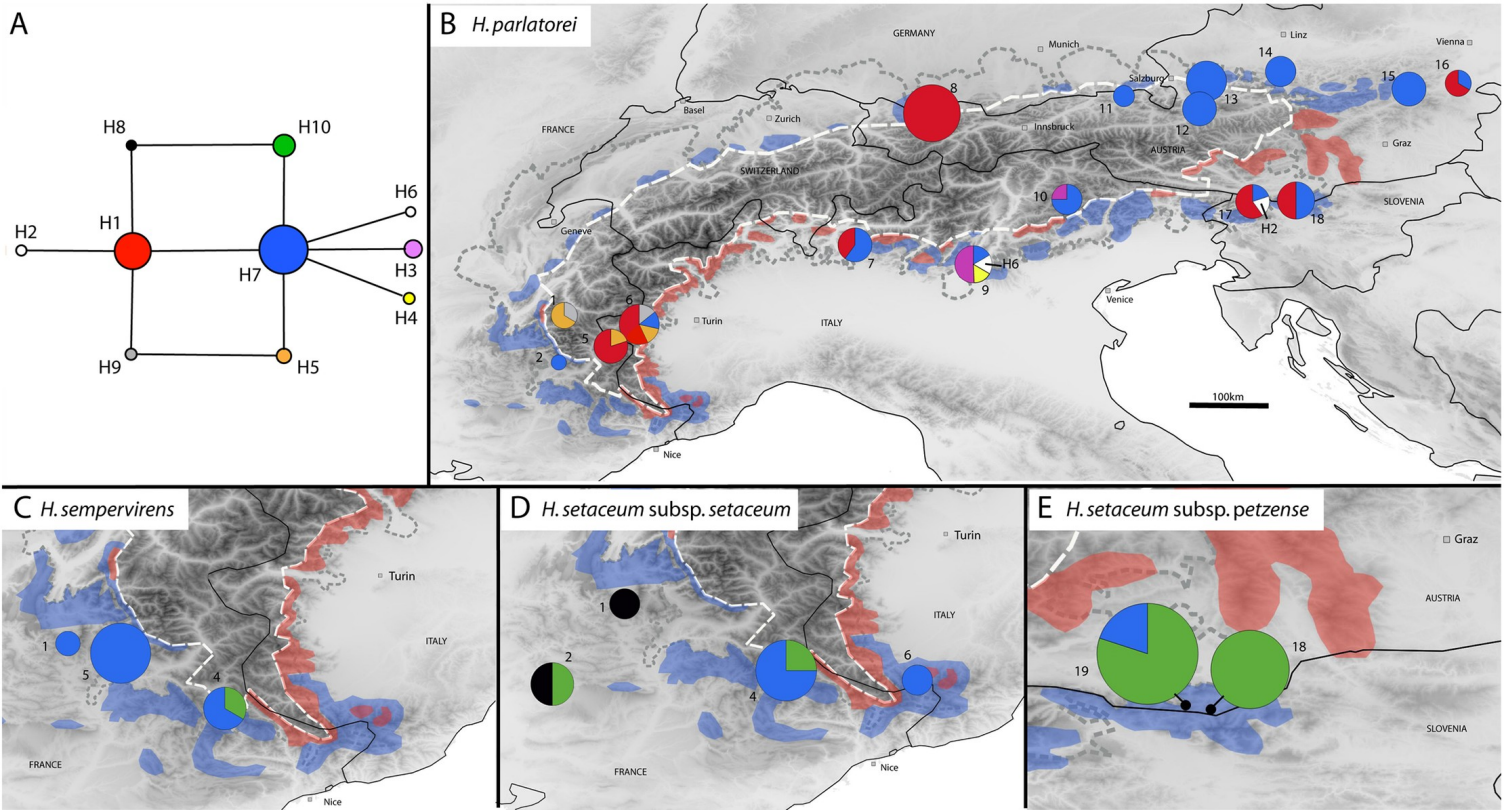
**Network (A) and geographic distribution of the 18 nDNA (*At103*) haplotypes (H1 to H18) found in the four taxa of the Alpine *Helictotrichon parlatorei* species group obtained by a maximum parsimony method based on a median joining algorithm (B–E).** In the network chart size increases with sample size from 1 to 50 and in the geographic maps from 1 to 14 for *H*. *parlatorei*, 1 to 6 for *H*. *sempervirens* and 1 to 4 *H*. *setaceum* subsp. *setaceum* and by 3 to 5 for subsp. *petzense* (see Table 2). See Fig 1 for further explanation. For population names by ID see S3 Table. Haplotypes in white are rare haplotypes occurring only in a single population.

In total 106 At103-sequences (81 *Helictotrichon parlatorei*, 7 *H*. *setaceum* subsp. *setaceum*, 8 subsp. *petzense* and 10 *H*. *sempervirens*) were obtained yielding an alignment of 320bp with 7 SNPs. These combined into 10 haplotypes (8 *H*. *parlatorei*, 3 *H*. *setaceum* subsp. *setaceum*, 2 subsp. *petzense* and 2 *H*. *sempervirens*) (Table 2). The shortest haplotype network needed seven mutations. Haplotypes were separated by one mutation (Fig 2). Some mutations were homoplasious resulting in loops. The most frequent and widely distributed haplotype was H7 found in all four species and across the entire Alps except for the central northern flank. It was quite dominant in the eastern region in *H*. *parlatorei* and in the Western Alps in *H*. *setaceum* subsp. *setaceum* and *H*. *sempervirens*. Also H1 was rather widespread but only found in *H*. *parlatorei*. All other haplotypes of *H*. *parlatorei* were rare and rather locally restricted. Six out of nine haplotypes were private in *H*. *parlatorei* with the center of haplotype diversity in the Western Alps. A further center of diversity was found in the Southern Alps. The eastern and northern populations exhibited rather low haplotype diversities. The level of population structure (G_ST_) in *H*. *parlatorei* was 0.3871 (p<0.001). There was a significant phylogeographic signal (N_ST_ = 0.4092; p<0.001) with closely related haplotypes found geographically close to each other; thus the N(d) curve was significantly negative (b-log = -0.1616; p < 0.001, S1 Fig) with a continuously decreasing curve. H8 and H10 were the only haplotypes absent in *H*. *parlatorei*. H10, however, was present in all narrow endemics of Alpine *Helictotrichon*. It was thus widely distributed along the southern flank of the Alps.

**Table 2:** Number of individuals per taxon bearing the haplotypes of the nuclear marker *At103* in the *Helictotrichon parlatorei* species group.

Haplotypes	*H*. *parlatorei*	*H*. *sempervirens*	*H*. *setaceum* subsp. *setaceum*	*H*. *setaceum* subsp. *petzense*	Total
**1**	30				30
**2**	2				2
**3**	4				4
**4**	1				1
**5**	4				4
**6**	1				1
**7**	37	9	3	1	50
**8**			2		2
**9**	2				2
**10**		1	2	7	10
**Total**	81	10	7	8	106

### Plastid DNA pattern

**Fig 3:**
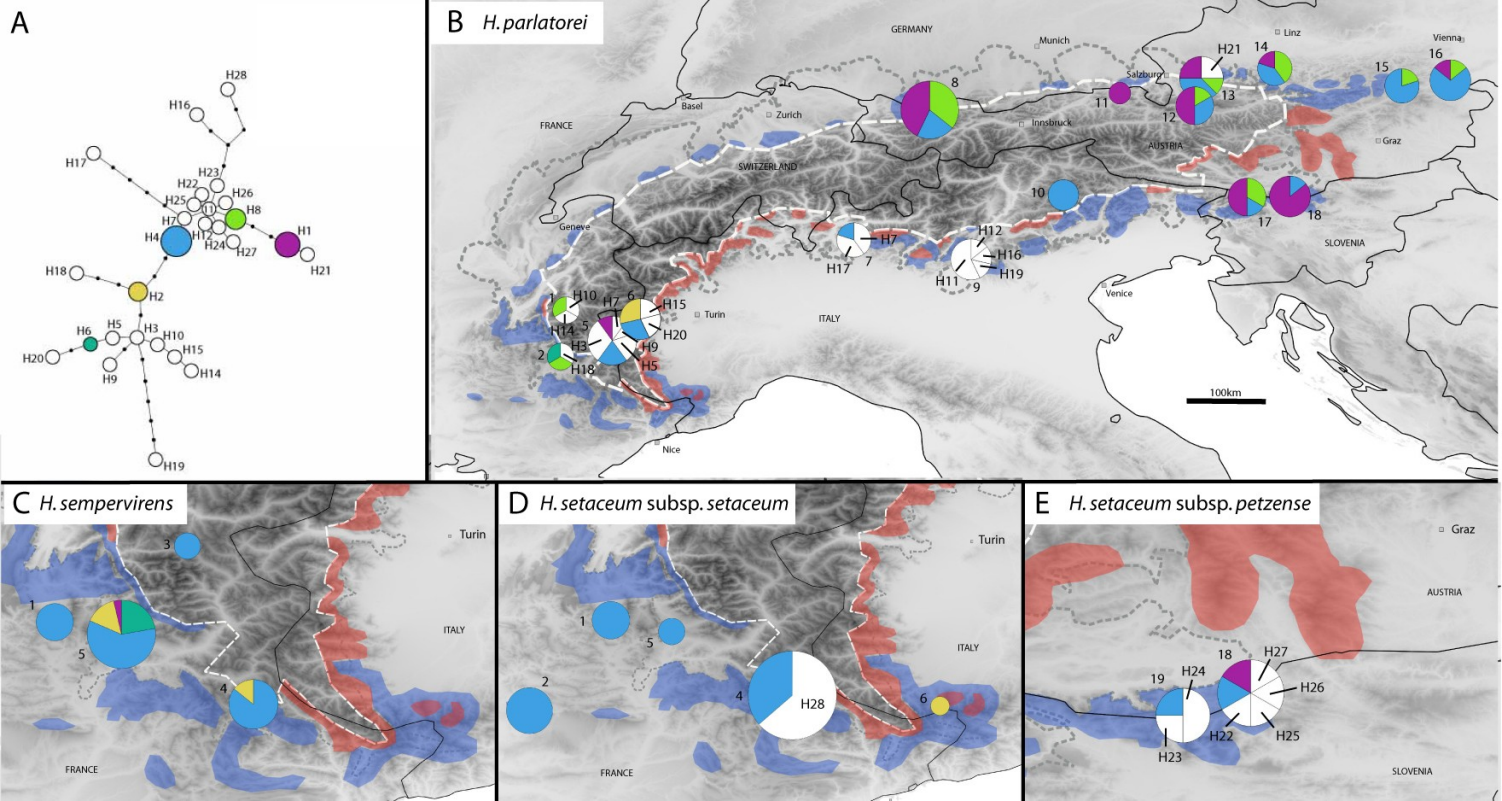
**Network (A) and geographic distribution of the 27 plastid DNA (*rps16*, *rpL*32–*trnL* (UAG), *ycf*3ln1, *ycf*3ln2) haplotypes (H1 to H28) found in the four taxa of the Alpine *Helictotrichon parlatorei* species group obtained by a maximum parsimony method based on a median joining algorithm (B—E).** In the network chart size increases with sample size from 1 to 50 and in the geographic maps from 2 to 14 for *H*. *parlatorei*, 1 to 8 for *H*. *sempervirens* and 1 to 5 *H*. *setaceum* subsp. *setaceum* and by 10 for subsp. *petzense* (Table 4). See Fig 1 for further explanation. For population names by ID see S3 Table. Haplotypes in white are rare haplotypes occurring only in a single population.

In total 485 sequences were obtained in four genetic markers for 133 individuals (Table 3). The final alignment of the supermatrix was 3,142bp long with 89 SNPs. These combined into 27 haplotypes (Table 4). The shortest haplotype network needed 50 mutations. 11 haplotypes clustered in the center of the network with one mutation between haplotypes (Fig 3). Four groups of 1 to 11 haplotypes connected loosely to this center at the haplotypes H4, H7, H8 and H23 with one to 6 mutations between adjacent haplotypes. The network contained three frequent haplotypes: H1, H4 and H8 are distributed across the entire Alps. While H4 was most frequent in *H*. *parlatorei* in the Eastern Alps and represented also the dominant haplotype in *H*. *sempervirens*, *H*. *setaceum* subspp. *setaceum* and *petzense* in the Western Alps, H1 and H8 were almost restricted to *H*. *parlatorei* in the Eastern Alps. Only by a single individual each of *H*. *parlatorei* and *H*. *sempervirens* it was present also in the Western Alps. All other haplotypes were rare.

**Table 3:** Details of the dataset of nuclear *At103* and the supermatrix of four plastid markers sequenced in the four taxa of the *Helictotrichon parlatorei* species group.

	*n DNA*	*p DNA*				
	*At103*	*rps16*	*rpl3-trnL*(UAG)	*ycf3*In1	*ycf3*In2	Total supermatrix
Alignment length (bp)	320	713	789	880	760	3142
# of SNPs	7	25	35	20	9	89
#of gaps	23	18	23	37	0	101
# of haplotypes	10	27	24	19	17	27
*H*. *parlatorei*	81	89	93	74	97	97
*H*. *sempervirens*	10	13	10	12	13	14
*H*. *setaceum* subsp. *setaceum*	7	11	9	9	11	11
*subsp*. *petzense*	8	10	10	10	8	10
Total # of sequences	106	124	122	110	129	133

#. Number.

**Table 4:** Number of individuals per taxon bearing the haplotypes of the combined plastid markers (*rps16*, *rpL*32-*trnL* (UAG), *ycf*3ln1, *ycf*3ln2) in *Helictotrichon parlatorei* species group.

Haplotypes	*H*. *parlatorei*	*H*. *sempervirens*	*H*. *setaceum* subsp. *setaceum*	*H*. *setaceum* subsp. *petzense*	Total
**1**	25	1		1	27
**2**	2	3	1		6
**3**	3				3
**4**	30	9	9	2	50
**5**	2				2
**6**	1	1			2
**7**	3				3
**8**	15				15
**9**	1				1
**10**	1				1
**11**	4				4
**12**	1				1
**14**	1				1
**15**	1				1
**16**	1				1
**17**	2				2
**18**	1				1
**19**	1				1
**20**	1				1
**21**	2				2
**22**				1	1
**23**				1	1
**24**				2	2
**25**				1	1
**26**				1	1
**27**				1	1
**28**			1		1
**Total**	97	14	11	10	133

In *H*. *parlatorei* the center of genetic diversity was found in the Western and Southern Alps. The level of population structure (G_ST_) was 0.1886 (p<0.001). There was no phylogeographic signal (N_ST_ = 0.1895; p<0.551) and overall the N(d) curve was only slightly negative (b-log = -0.052; p = 0.029, S2 Fig). This was due to a high peak at 300 km which was equivalent to the extent of the isolated region of the Western Alps and its distance to populations in the Eastern Alps so that only at distances above 300 km individuals of Western and Eastern Alps were compared (S2 Fig). There are no genetic differences between di- and tetraploids in *H*. *parlatorei*. The respective individuals showed the same nuclear and plastid haplotypes as any other individual.

The genetic diversity of *H*. *sempervirens* and *H*. *setaceum* subsp. *setaceum* was very low compared to *H*. *parlatorei* in the Western Alps. In contrast, *H*. *setaceum* subsp. *petzense* in the southeastern Alps was much more diverse than *H*. *sempervirens* and *H*. *setaceum* subsp. *setaceum* in the Western Alps and *H*. *parlatorei* in that same area of the Southeastern Alps.

## Discussion

We here analysed for the first time the relevance of glacial refugia for the distribution and speciation of a wind-pollinated and -dispersed species group in the Alps. Although the current distribution patterns of the species are widely congruent with the regions of the Alps which remained unglaciated during the last glacial period, the phylogeographic pattern does not suggest all of those areas to have harbored these species also during glaciation. Instead only the Western, Central Southern and some Southeastern populations of the Alps exhibit elevated genetic diversity suggesting those areas to have served as refugia. The entire Eastern Alps are overall rather genetically depauperate indicating a recent colonization via efficient long distance wind dispersal.

### Species delimitation

Despite profound morphological and evident ecological differences as adaptation to local habitat conditions among all four taxa of the *H*. *parlatorei* species group [16] there is rather low genetic differentiation among the species. There are only a few private haplotypes per species (Tables 2 and 4). Instead a lot of genetic information is still shared between all species (e.g. nuclear DNA: At103-H7; plastid DNA: H4). This indicates either a recent speciation and/or an ongoing gene flow between species. It is difficult to distinguish between both mechanisms here, as the distribution ranges and collection sizes of *H*. *setaceum* subsp. *setaceum* and subsp. *petzense* and *H*. *sempervirens* are very small and spatially restricted, making it impossible to decide whether plastid haplotypes are distributed at random (ancestral polymorphism) or follow concordant geographic patterns across species (hybridisation) [36, 37]. Detailed morphological field-investigations indicate the presence of morphological intermediates between *H*. *parlatorei* and *H*. *setaceum* subsp. *petzense* and thus ongoing hybridisation between those two taxa [21, 27]. To confirm those results we suggest enlarging the current sampling of individuals and independent loci.

For *H*. *sempervirens* earlier karyological studies suggest a hybrid origin from the parental species *H*. *parlatorei* and *H*. *setaceum* [23]. This is supported here as *H*. *sempervirens* is the only species whose genetic diversity (nuclear and plastid) is based on the presence of haplotypes from both *H*. *parlatorei* and the two geographically restricted subspecies of *H*. *setaceum* and not on private haplotypes on its own as in the case of *H*. *setaceum* subsp. *setaceum* (nuclear haplotype: H8) and subsp. *petzense* (6 private plastid haplotypes) (Tables 2 and 4). The genetic species delimitation of the *H*. *parlatorei* species group has never been tested before as in earlier phylogenies only a single individual per species was used [22].

### Intraspecific differentiation–origin and migration

Despite a disjunct distribution of *H*. *parlatorei* across the Alps with major gaps in the Swiss Alps and the central Eastern Alps no equivalently separated gene pools could be detected. Instead in both genomes (nuclear and plastid) haplotypes were widespread across the whole distribution range (Figs 2 and 3). The absence of congruent gene pools indicates effective dispersal of this species across its entire distribution area—largely unaffected by the alpine topography. A similar lack of genetic differentiation across large distances can repeatedly be found in phylogeographic studies of other wind-pollinated grass species [38–40]. Only in the plastid genome, which directly reflects seed dispersal due to its predominantly maternal inheritance [41], the West-East disjunction in the Alps becomes a little apparent by an accumulation of numerous rare haplotypes solely in the Western Alps producing a considerable peak in the N(d) curve at 300km (S2 Fig). In the biparentally inherited n-genome [42–44] a weak phylogeographic structure was present, however, without such a remarkable unequal distribution of rare haplotypes between Western and Eastern Alps as with the plastid genome. This indicates slightly more efficient long distance dispersal via pollen than seeds. Thus as most wind-pollinated species of the Alps also the species of the *H*. *parlatorei* species group show an adaptation to dryer environments at lower altitudes of the montane to subalpine zone, which is rather advantageous for wind dispersal [45]. The species thus escape the more stressful conditions of lower temperature, higher precipitation and longer snowcover at higher altitude [46].

The center of genetic diversity in *H*. *parlatorei* is found in the Western and central Southern Alps. Thus these phylogeographic patterns suggest the survival of *H*. *parlatorei* in a maximum of two refugia at the western and central southern periphery of the Alps. This is in sharp contrast with findings in other alpine herbs which show a strong genetic structure between populations from different parts of the Alps indicating a survival in several refugia all around the Alps with little expansion at climate amelioration (see [6]). In accordance with prior phylogeographic studies the expansion follows the presence of calcareous bedrock to the Eastern Alps [8]. Also the absence of *H*. *parlatorei* in the Swiss Alps and the central Eastern Alps is based on the presence of siliceous bedrock here and corresponds with major biogeographic boundaries in the areas (see [6] and Fig 1). The absence along the northern flank of the Swiss Alps with limestone, however, remains unexplained.

The sampling of the other taxa of *Helictotrichon* studied is too small to identify areas of geographic origin and expansion. Here a larger sampling would be necessary. Based on morphological similarities Melzer [27], however, hypothesized that an ancestor of *H*. *setaceum* once had a larger distribution with only two remnant survivals in the Southern Alps, which developed into *H*. *petzense* in the Southeast and *H*. *setaceum* in the West. Indeed, the genetic affinity in the nuclear genome might support a closer relationship of *H*. *petzense* to *H*. *setaceum* than to *H*. *parlatorei* despite a far larger geographic distance between the former two species. On morphological grounds, *H*. *petzense* and *H*. *setaceum* were previously merged as two subspecies under a single species [17].

The comparisons of genetic diversity among species in the same area each revealed marked differences. Here differences in biological traits between species might play a role [47, 48], however, to confirm this more detailed ecological studies would be necessary in the *Helictotrichon* species.

### A speciation scenario

Based on the identified centers of genetic diversity, the deduced migration routes and morphological comparisons we come up with the following speciation scenario for the *H*. *parlatorei* species group:

The common ancestor of the four *Helictotrichon* taxa originates probably in the current diversity center of the *H*. *parlatorei* species group in the Western Alps. Unfortunately, a further sister group support for this geographic placement of its origin is still not available as only the monophyly of the *H*. *parlatorei* species group is supported by plastid and nuclear ribosomal DNA [22, 24] but its sister group is not yet unambiguously identified. Potential sisters occur in the Northeast of the Alps (continental *H*. *desertorum*), the southeastern edge of the Alps (Mediterranean *H*. *convolutum*) and the Western Alps (orophytic west Mediterranean *H*. *sedenense*) [17].

Starting in the Western Alps we thus hypothesize a consecutive sympatric ecological speciation of *H*. *parlatorei* and *H*. *setaceum* with *H*. *parlatorei* adapting to montane/subalpine screes and dwarf-shrub communities and *H*. *setaceum* to thermophilic and chasmophytic plant communities on calcareous rock [16]. A similar ecological divergence can be found among the Alpine species of several plant genera (e.g. *Phyteuma* s.l.; [49]). From the Western Alps, potentially both *Helictotrichon* species dispersed towards the Eastern Alps. A once wider distribution of *H*. *setaceum* along the Southern Alps and subsequent fragmentation of this range during the glacial cycles (vicariance) led to an eastern relict population, which slowly diverged in isolation and now represents subsp. *petzense* that survived only on the rare habitat of almost vertical and bare rocks in the Southeastern Alps and shows a remarkable accumulation of rare plastid haplotypes. This fragmentation of a once continuous range seems more likely than long-distance dispersal across the almost entire Alpine Arc. Consequently, *Helictotrichon parlatorei* is the only widespread species in the Alps today, which might be due to a higher cold resistance of this species [16]. The third species from the Western Alps, hexaploid *H*. *sempervirens*, must have established later through hybridisation of *H*. *parlatorei* and *H*. *setaceum* subsp. *setaceum* in their area of distributional overlap as established through sequencing of the nuclear single copy gene topoisomerase 6 and cytological investigations [24, 50].

## Supporting information

S1 Table Haplotype (Hap) ID, voucher information and GenBank accession numbers of all haplotypes found in the Alpine *Helictotrichon* (Poaceae). H, Herbarium code; nd, no data.
(DOCX)

S2 Table Voucher information of all sampled accessions of Alpine *Helictotrichon* used in this analysis. A–Austria, D–Germany, I–Italy, F–France, SLO–Slovenia, #NV–no data.
(DOCX)

S3 Table Sampled populations for the phylogeographic analyses of the Alpine *Helictotrichon* species. NE, Northeast; NW, Northwest.
(DOCX)

S1 Fig Average kinship N(d) between individuals of *Helictotrichon parlatorei* according to geographical distances based on nuclear sequences (*At103*). Kinship coefficients between individuals are standardized relative to the unweighted average allele frequencies over species.
(DOCX)

S2 Fig Average kinship N(d) between individuals of *Helictotrichon parlatorei* according to geographical distances based on combined chloroplast sequences (*rps16, rpL*32-*trnL* (UAG), *ycf*3ln1, *ycf*3ln2). Kinship coefficients between individuals are standardized relative to the unweighted average allele frequencies over species.
(DOCX)
