# Supplementary material for: Glacial refugia and speciation in a group of wind-pollinated and -dispersed, endemic Alpine species of Helictotrichon (Poaceae)
Source: PLoS One. 2018 Oct 15;13(10):e0205354. doi: 10.1371/journal.pone.0205354 (PMC6188759; doi:10.1371/journal.pone.0205354)
Supplement: S1 Table — H, Herbarium code; nd, no data. (DOCX) [file pone.0205354.s001.docx]

**Supporting Information**

**S1 Table.** **Haplotype (Hap) ID, voucher information and GenBank accession numbers of all haplotypes found in the Alpine *Helictotrichon* (Poaceae).** H, Herbarium code; nd, no data.

|  |  |  |  |  | **Genbank Accession Number** | | | | |
| --- | --- | --- | --- | --- | --- | --- | --- | --- | --- |
| **Hap_ID** | **Species** | **Collector** | **Coll.-Nb.** | **H** | ***At103*** | ***rps16*** | ***rpl3-trnL*(UAG)** | ***ycf3*In1** | ***ycf3*In2** |
| 1 |  |  |  |  | MH156681 |  |  |  |  |
| 2 |  |  |  |  | MH156682 |  |  |  |  |
| 3 |  |  |  |  | MH156683 |  |  |  |  |
| 4 |  |  |  |  | MH156684 |  |  |  |  |
| 5 |  |  |  |  | MH156685 |  |  |  |  |
| 6 |  |  |  |  | MH156686 |  |  |  |  |
| 7 |  |  |  |  | MH156687 |  |  |  |  |
| 8 |  |  |  |  | MH156688 |  |  |  |  |
| 9 |  |  |  |  | MH156689 |  |  |  |  |
| 10 |  |  |  |  | MH156690 |  |  |  |  |
|  |  |  |  |  |  |  |  |  |  |
| 1 |  |  |  |  |  | MH090990 | MH156717 | MH156745 | MH223673 |
| 2 |  |  |  |  |  | MH090983 | MH156710 | MH156738 | MH223666 |
| 3 |  |  |  |  |  | MH090987 | MH156714 | MH156742 | MH223670 |
| 4 |  |  |  |  |  | MH090988 | MH156715 | MH156743 | MH223671 |
| 5 |  |  |  |  |  | MH090984 | MH156711 | MH156739 | MH223667 |
| 6 |  |  |  |  |  | MH090980 | MH156707 | MH156735 | MH223663 |
| 7 |  |  |  |  |  | MH090985 | MH156712 | MH156740 | MH223668 |
| 8 |  |  |  |  |  | MH090972 | MH156699 | MH156727 | MH223655 |
| 9 |  |  |  |  |  | MH090986 | MH156713 | MH156741 | MH223669 |
| 10 |  |  |  |  |  | MH090989 | MH156716 | MH156744 | MH223672 |
| 11 |  |  |  |  |  | MH090964 | MH156691 | MH156719 | MH223647 |
| 12 |  |  |  |  |  | MH090965 | nd | MH156718 | MH223646 |
| 13 |  |  |  |  |  | MH090966 | MH156692 | MH156720 | MH223648 |
| 14 |  |  |  |  |  | MH090967 | MH156693 | MH156721 | MH223649 |
| 15 |  |  |  |  |  | MH090981 | MH156708 | MH156736 | MH223664 |
| 16 |  |  |  |  |  | MH090968 | MH156694 | MH156722 | MH223650 |
| 17 |  |  |  |  |  | MH090969 | MH156695 | MH156723 | MH223651 |
| 18 |  |  |  |  |  | MH090970 | MH156696 | MH156724 | MH223652 |
| 19 |  |  |  |  |  | MH090971 | MH156697 | MH156725 | MH223653 |
| 20 |  |  |  |  |  | MH090973 | MH156698 | MH156726 | MH223654 |
| 21 |  |  |  |  |  | MH090974 | MH156700 | MH156728 | MH223656 |
| 22 |  |  |  |  |  | MH090975 | MH156701 | MH156729 | MH223657 |
| 23 |  |  |  |  |  | MH090976 | MH156702 | MH156730 | MH223658 |
| 24 |  |  |  |  |  | MH090977 | MH156703 | MH156731 | MH223659 |
| 25 |  |  |  |  |  | MH090978 | MH156704 | MH156732 | MH223660 |
| 26 |  |  |  |  |  | MH090979 | MH156705 | MH156733 | MH223661 |
| 27 |  |  |  |  |  | MH090982 | MH156706 | MH156734 | MH223662 |
| 28 |  |  |  |  |  | MH090990 | MH156709 | MH156737 | MH223665 |
