# Supplementary material for: Glacial refugia and speciation in a group of wind-pollinated and -dispersed, endemic Alpine species of Helictotrichon (Poaceae)
Source: PLoS One. 2018 Oct 15;13(10):e0205354. doi: 10.1371/journal.pone.0205354 (PMC6188759; doi:10.1371/journal.pone.0205354)
Supplement: S2 Table — A–Austria, D–Germany, I–Italy, F–France, SLO–Slovenia, #NV–no data. (DOCX) [file pone.0205354.s002.docx]

**Supporting Information**

**S2 Table. Voucher information of all sampled accessions of Alpine *Helictotrichon* used in this analysis.** A – Austria, D – Germany, I – Italy, F – France, SLO – Slovenia, #NV – no data.

| **Provenance** | **Latitude** | **Longitude** | **Collector, collection number, collection date** | **Herbarium** | | **Laboratory DNA extraction number** | | **Geographical grouping in this study** | | **nHap-ID** | | **cpHap-ID** | |  |
| --- | --- | --- | --- | --- | --- | --- | --- | --- | --- | --- | --- | --- | --- | --- |
| ***Helictotrichon parlatorei* (J. Woods) Pilger** | | | | | | | | | | | | | |  |
| A, Carinthia, Hochstuhl | 46.43402 N | 14.17387 E | P. Gutte 244/2000, 29.06.2000 | | LZ 162916 | | 1105 | | Karawanks | | 7 | | 1 | |
| A, Karawanks, Dicke Koschuta | 46.44694 N | 14.42944 E | P. Gutte & W. Morawetz 204/97, 09.07.1997 | | LZ 157772 | | 1103 | | Karawanks | | 7 | | 1 | |
| A, Karawanks, Petzen | 46.50972 N | 14.77000 E | M. Röser 10648, 10.07.1998 | | HAL | | 198 | | Karawanks | | 1 | | 1 | |
| A, Karawanks, Petzen | 46.51083 N | 14.76917 E | B. Heuchert 401, 02.08.2001 | | HAL | | 854 | | Karawanks | | 1 | | 1 | |
| A, Karawanks, Petzen | 46.51000 N | 14.77222 E | B. Heuchert 1101, 03.08.2001 | | HAL | | 855 | | Karawanks | | 1 | | 4 | |
| A, Karawanks, Petzen | 46.51000 N | 14.77222 E | B. Heuchert 1105, 03.08.2001 | | HAL | | 895 | | Karawanks | | #NV | | 1 | |
| A, Karawanks, Petzen | 46.50417 N | 14.75861 E | P. Gutte & W. Morawetz 224/97, 11.07.1997 | | LZ 157812 | | 1104 | | Karawanks | | 7 | | 1 | |
| A, Lower Austria, Schneeberg | 47.75389 N | 15.83750 E | B. Wallnöfer s.n., s.d. | | W | | 1159 | | Schneeberg | | 7 | | 4 | |
| A, Lower Austria, Schneeberg | 47.75389 N | 15.83750 E | B. Heuchert 1806, 06.08.2001 | | HAL | | 856 | | Schneeberg | | #NV | | 4 | |
| A, Lower Austria, Schneeberg | 47.75722 N | 15.83611 E | B. Heuchert 1501, 06.08.2001 | | HAL | | 857 | | Schneeberg | | 1 | | 4 | |
| A, Lower Austria, Schneeberg | 47.75722 N | 15.83611 E | B. Heuchert 1502, 06.08.2001 | | HAL | | 892 | | Schneeberg | | 1 | | 4 | |
| A, Lower Austria, Schneeberg | 47.75389 N | 15.83750 E | B. Heuchert 1804, 06.08.2001 | | HAL | | 893 | | Schneeberg | | #NV | | 8 | |
| A, Lower Austria, Schneeberg | 47.75389 N | 15.83750 E | B. Heuchert 1801, 06.08.2001 | | HAL | | 894 | | Schneeberg | | #NV | | 4 | |
| A, Lower Austria, Waxriegel | 47.76000 N | 15.83028 E | M. Röser 12120, 04.08.1986 | | HAL | | 847 | | Schneeberg | | #NV | | 1 | |
| A, Salzburg, Benzegg | 47.26389 N | 13.39472 E | P. Pilsl s.n., 11.08.2013 | | SZU 23399 | | 1099 | | Salzburg | | 7 | | 4 | |
| A, Salzburg, Filzmoos | 47.46861 N | 13.56472 E | P. Pilsl s.n., 26.07.2014 | | SZU 24347 | | 1102 | | Salzburg | | 7 | | 1 | |
| A, Salzburg, Korein-Gipfel | 47.46690 N | 13.35468 E | P. Pilsl s.n., 08.08.2004 | | SZU 14246 | | 1097 | | Salzburg | | 7 | | 1 | |
| A, Salzburg, Rauriser Tal | 47.24611 N | 13.02000 E | P. Pilsl s.n., 13.07.2010 | | SZU 20774 | | 1100 | | Salzburg | | 7 | | 4 | |
| A, Salzburg, Schafberg | 47.77639 N | 13.43361 E | H. Wagner s.n., 19.06.1987 | | SZU 24507 | | 1154 | | Salzburg | | #NV | | 1 | |
| A, Salzburg, Trattberg | 47.64083 N | 13.27389 E | H. Wittmann s.n., 21.07.1990 | | LI 044097 | | 1117 | | Salzburg | | 7 | | 8 | |
| A, Salzkammergut, Graskögerl | 47.70278 N | 15.09472 E | H. Melzer s.n., 21.06.1983 | | LI 780870 | | 1114 | | Salzkammergut | | 7 | | 21 | |
| A, Salzkammergut, Hoher Plassen | 47.57111 N | 13.61361 E | B. Wallnöfer 14728, 05.07.2012 | | W | | 1145 | | Salzkammergut | | 7 | | 1 | |
| A, Salzkammergut, Leonsberg | 47.75444 N | 13.56889 E | P. Pilsl s.n., 24.07.1993 | | SZU 9277 | | 1101 | | Salzkammergut | | 7 | | 4 | |
| A, Salzkammergut, Schafberg | 47.77678 N | 13,43065 E | P. Pilsl s.n., 01.07.2000 | | SZU 11235 | | 1098 | | Salzkammergut | | 7 | | 8 | |
| A, Styria, Admont | 47.53333 N | 14.51667 E | C. Scheuer s.n., 01.07.1992 | | GZU 000321997 | | 1108 | | Salzkammergut | | 7 | | 4 | |
| A, Styria, Bärenloch | 47.49833 N | 13.74944 E | A. Drescher s.n., 04.06.1994 | | GZU 210587 | | 1107 | | Salzkammergut | | 7 | | 4 | |
| A, Styria, Dachstein Mountains | 47.50222 N | 13.98056 E | K. Zernig 5293, 02.06.2007 | | GJO 27.281/25 | | 1109 | | Salzkammergut | | 7 | | 1 | |
| A, Styria, Gesäuse | 47.54532 N | 14.57276 E | M. Strauch s.n., 00.08.1991 | | LI 568972 | | 1119 | | Salzkammergut | | #NV | | 4 | |
| A, Styria, Mürzsteg Alps | 47.65000 N | 15.40000 E | K. Zernig 6416, 22.07.2009 | | GJO 27.451/4 | | 1110 | | Eisenerzer Alpen | | 7 | | 4 | |
| A, Styria, Rax | 47.69000 N | 15.69000 E | M. Krusche 64/2000, 18.06.2000 | | LZ 162764 | | 1096 | | Eisenerzer Alpen | | 7 | | 4 | |
| A, Styria, Rax, Schlangenweg | 47.68203 N | 15.70633 E | B. Wallnöfer s.n., 11.07.2015 | | W | | 1146 | | Eisenerzer Alpen | | 7 | | 4 | |
| A, Styria, Tauplitz | 47.58227 N | 14.00388 E | H. Melzer s.n., 19.06.1993 | | LI 150329 | | 1116 | | Salzkammergut | | 7 | | 21 | |
| A, Styria, Trechtling | 47.53194 N | 15.00972 E | H. Melzer s.n., 28.06.1992 | | GZU 210440 | | 1106 | | Eisenerzer Alpen | | 7 | | 8 | |
| A, Tyrol, Brennhütte | 47.61833 N | 12.54861 E | S. Kattari 4548 & W. Lippert, A. Schmidt, F. Dunkel, 26.07.2013 | | M 0222068 | | 1095 | | Tyrol | | 7 | | 1 | |
| A, Tyrol, Fellhorn | 47.60972 N | 12.50861 E | S. Kattari 4561 & S. Unterhuber, 03.08.2013 | | M 0222067 | | 1094 | | Tyrol | | 7 | | 1 | |
| A, Upper Austria, Almkogel | 47.80556 N | 13.36528 E | V. Zila s.n., 19.07.1992 | | LI 320830 | | 1113 | | Upper Austria | | 7 | | 4 | |
| A, Upper Austria, Brunnstein | 47.61667 N | 14.30000 E | G. Pils s.n., 31.07.1991 | | KL 146661 | | 1150 | | Upper Austria | | #NV | | 1 | |
| A, Upper Austria, Großer Phrygas | 47.64889 N | 14.39167 E | G. Kleesadl 2986, 13.07.2000 | | LI 435684 | | 1120 | | Upper Austria | | 7 | | 8 | |
| A, Upper Austria, Grünberg | 48.34833 N | 14.67889 E | M. Strauch s.n., 09.05.1994 | | LI 167882 | | 1115 | | Upper Austria | | 7 | | 4 | |
| A, Upper Austria, Molln | 47.88583 N | 14.25694 E | M. Strauch s.n., 07.06.1993 | | LI 235384 | | 1112 | | Upper Austria | | 7 | | 8 | |
| D, Bavaria, Community Farchant | 47.54139 N | 11.16222 E | F. Schuhwerk 92/237, 28.07.1992 | | M 0222056 | | 1055 | | Bavaria | | 1 | | 1 | |
| D, Bavaria, Ettaler Mandl | 47.58556 N | 11.11722 E | W. Kortenhaus 2d, 02.07.1997 | | M 0222060 | | 1059 | | Bavaria | | 1 | | 8 | |
| D, Bavaria, Fricken | 47.53270 N | 11.15466 E | R. Urban 234, 24.06.1997 | | M 0222061 | | 1060 | | Bavaria | | 1 | | 8 | |
| D, Bavaria, Geißsprungkopf | 47.54019 N | 10.99965 E | A. Mayer 20, 02.07.1996 | | M 0222059 | | 1058 | | Bavaria | | 1 | | 1 | |
| D, Bavaria, Grenzsattel | 47.53806 N | 10.55667 E | E. Dörr s.n., 17.07.1999 | | M 0222062 | | 1061 | | Bavaria | | 1 | | 1 | |
| D, Bavaria, Heimgarten | 47.61361 N | 11.28194 E | M. Wecker 18, 23.07.1997 | | M 0222063 | | 1062 | | Bavaria | | 1 | | 8 | |
| D, Bavaria, Herzogstand | 47.61625 N | 11.29963 E | W. Lippert 29132, 03.07.2006 | | M 0222065 | | 1064 | | Bavaria | | 1 | | 1 | |
| D, Bavaria, Koblat | 47.41998 N | 10.36749 E | U. Kohler 04/140, 31.07.2004 | | M 0222053 | | 1052 | | Bavaria | | 2 | | 1 | |
| D, Bavaria, Notkarspitze | 47.55361 N | 11.05500 E | F. Eberlein s.n., 14.06.2000 | | M 0222064 | | 1063 | | Bavaria | | 1 | | 4 | |
| D, Bavaria, Oberauer Steig | 47.54163 N | 11.15189 E | A. Saitner s.n., 09.–10.08.1997 | | M 0222054 | | 1053 | | Bavaria | | 1 | | 8 | |
| D, Bavaria, Osterfelder Kopf | 47.44720 N | 11.06177 E | P. Gutte & X. Menhofer s.n., 20.08.1991 | | LZ 46291 | | 1065 | | Bavaria | | 1 | | 1 | |
| D, Bavaria, Osterfelderkopf | 47.43900 N | 11.04996 E | H. Wagner s.n., 05.07.1989 | | SZU 26122 | | 1051 | | Bavaria | | #NV | | #NV | |
| D, Bavaria, Ridge Heimgarten-Herzogstand | 47.61384 N | 11.30730 E | A. Mayer 107, 02.07.1993 | | M 0222057 | | 1056 | | Bavaria | | 1 | | 4 | |
| D, Bavaria, Soiernspitze | 47.47572 N | 11.35690 E | R. Urban 26, 1994 | | M 0222058 | | 1057 | | Bavaria | | 1 | | 8 | |
| D, Bavaria, Ziegelspitz | 47.55467 N | 11.06972 E | A. Lang s.n., 18.08.1998 | | M 0222055 | | 1054 | | Bavaria | | 1 | | 4 | |
| F, Cottian Alps, Col Perdue | 45.40587 N | 7.18457 E | H. Teppner s.n., 03.07.1964 | | LI 571984 | | 1118 | | Hautes-Alpes | | #NV | | 1 | |
| F, Cottian Alps, Pic de Rochebrune | 44.82250 N | 6.78722 E | G. Pils 261, 28.07.1990 | | KL 119683 | | 1152 | | Hautes-Alpes | | 5 | | 3 | |
| F, Dépt. Hautes-Alpes, Col de Montgenèvre | 44.93167 N | 6.72553 E | M. Röser 2342, 15.08.1984 | | HAL | | 851 | | Hautes-Alpes | | #NV | | 4 | |
| F, Dépt. Hautes-Alpes, Col de Montgenèvre | 44.93167 N | 6.72553 E | M. Röser 2342, 15.08.1984 | | HAL | | 890 | | Hautes-Alpes | | #NV | | 3 | |
| F, Dépt. Hautes-Alpes, Mont Dauphin | 44.79139 N | 6.62579 E | M. Röser 2290, 12.08.1984 | | HAL | | 845 | | Hautes-Alpes | | #NV | | 7 | |
| F, Dépt. Hautes-Alpes, Mont Dauphin | 44.79139 N | 6.62579 E | M. Röser 2290, 12.08.1984 | | HAL | | 891 | | Hautes-Alpes | | 1 | | 5 | |
| F, Dépt. Hautes-Alpes, Mont Viso | 44.70441 N | 7.03974 E | M. Röser 2266, 11.08.1984 | | HAL | | 846 | | Hautes-Alpes | | 1 | | 3 | |
| F, Dépt. Hautes-Alpes, Ristoles | 44.76667 N | 6.95000 E | A. Charpin s.n., s.d. | | G 00428388 | | 1160 | | Hautes-Alpes | | #NV | | 4 | |
| F, Dépt. Hautes-Alpes, Valley Mélezet | 44.46491 N | 6.42746 E | M. Röser 2267, 11.08.1984 | | HAL | | 844 | | Hautes-Alpes | | 1 | | 9 | |
| F, Dépt. Hautes-Alpes, Valley Réallon | 44.60186 N | 6.35816 E | M. Röser 2408, 21.08.1984 | | HAL | | 832 | | Hautes-Alpes | | 1 | | 5 | |
| F, Graian Alps | 45.31769 N | 7.03022 E | H. Melzer s.n., 26.07.1966 | | LI 633792 | | 1083 | | Savoie | | #NV | | 8 | |
| F, Grand Lac | 46.56556 N | 5.74917 E | G. Pils 235, 25.07.1995 | | KL 119842 | | 1151 | | Savoie | | 7 | | 6 | |
| F, Maritime Alps, Monte Chajol | 44.10292 N | 7.52884 E | G. Schneeweiß, P. Schönswetter & A. Tribsch s.n., 08.07.1998 | | WU 082374 | | 1079 | | Maritime Alps | | 5 | | 8 | |
| F, Maritime Alps, Colle di Tenda | 44.15009 N | 7.56121 E | M. Röser 6642, 19.07.1989 | | HAL | | 852 | | Maritime Alps | | 9 | | 10 | |
| F, Maritime Alps, Tende | 44.11346 N | 7.522416 E | G. Schneeweiß, P. Schönswetter & A. Tribsch s.n., 08.07.1998 | | WU 082373 | | 1078 | | Maritime Alps | | 5 | | 14 | |
| F, Savoie, Mont Bochor | 45.38206 N | 6.72123 E | W. Greuter s.n., 27.07.1966 | | LI 716146 | | 1084 | | Savoie | | #NV | | 18 | |
| I, Cima Valdritta, Monte Baldo | 45.72639 N | 10.84389 E | P. Gutte 323/02, 01.07.2002 | | LZ 166721 | | 1076 | | Monte Baldo | | 3 | | 11 | |
| I, Dolomites, Passo Fedaia | 46.45875 N | 11.87547 E | H. Wittmann s.n., 15.07.2004 | | LI 561335 | | 1091 | | NE Italy | | 7 | | 4 | |
| I, Dolomites, Passo Fedaia | 46.44948 N | 11.87086 E | H. Wittmann s.n., 15.07.2004 | | LI 561038 | | 1092 | | NE Italy | | 7 | | 4 | |
| I, Norditalien, Val Canale | 46.50574 N | 13.31827 E | H. Melzer s.n., 27.10.1999 | | LI 780782 | | 1086 | | 0 | | #NV | | #NV | |
| I, Piedmont, Limone | 44.20000 N | 7.56667 E | G. Pils 320, 25.05.1995 | | KL 119944 | | 1153 | | NW Italy | | 5 | | 15 | |
| I, Prov. Aosta, Grajische Alpen | 45.54083 N | 7.01806 E | H. Wittmann s.n., 05.08.1998 | | LI 562971 | | 1089 | | NW Italy | | 7 | | 20 | |
| I, Prov. Bergamo, Bergamasque Alps | 45.90633 N | 9.38442 E | H. Wittmann s.n., 12.08.1996 | | LI 339574 | | 1081 | | Lombardy | | 7 | | 17 | |
| I, Prov. Bergamo, Lago di Lecco | 45.95343 N | 9.38773 E | H. Wittmann s.n., 11.08.1996 | | LI 339587 | | 1082 | | Lombardy | | 7 | | 17 | |
| I, Prov. Bergamo, M. Alben | 45.86222 N | 9.78194 E | M. Röser 10408, 30.07.1996 | | HAL | | 848 | | Lombardy | | 1 | | 4 | |
| I, Prov. Bergamo, M. Arera | 45.94477 N | 9.82276 E | M. Röser 10431, 01.08.1996 | | HAL | | 833 | | Lombardy | | 1 | | 7 | |
| I, Prov. Como, Zucco di Pesciola | 45.95361 N | 9.51222 E | H. Wittmann s.n., 19.07.2005 | | LI 588016 | | 1090 | | Lombardy | | 7 | | 7 | |
| I, Prov. Pordenone | 46.38135 N | 12.48990 E | F. Boscutti s.n., 30.07.2007 | | MFU 18952 | | 1138 | | NE Italy | | 7 | | 4 | |
| I, Prov. Pordenone, Monte Borga | 46.29278 N | 12.34917 E | M. Buccheri s.n., 13.06.2007 | | MFU 18953 | | 1137 | | NE Italy | | 3 | | 4 | |
| I, Prov. Torino, Colle delle Finestre | 45.06494 N | 7.01254 E | M. Röser 6530, 16.07.1989 | | HAL | | 850 | | NW Italy | | 1 | | 4 | |
| I, Prov. Torino, Fenestrelle | 45.08060 N | 7.06250 E | M. Röser 2346, 15.08.1984 | | HAL | | 849 | | NW Italy | | 1 | | 2 | |
| I, Prov. Torino, Monte Albergian (Gran Costa) | 45.03743 N | 7.02473 E | M. Röser 2344, 15.08.1984 | | HAL | | 658 | | NW Italy | | 1 | | 2 | |
| I, Prov. Torino, Monte Viso | 44.70314 N | 7.14317 E | M. Röser 6568, 17.07.1989 | | HAL | | 853 | | NW Italy | | 9 | | 4 | |
| I, Prov. Trento, Monte Baldo | 45.73101 N | 10.84368 E | Institute excursion s.n., 05.06.1982 | | SZU 17342 | | 1074 | | Monte Baldo | | #NV | | 11 | |
| I, Prov. Verona, Monte Baldo | 45.73379 N | 10.83423 E | G. van Buggenhout s.n., 07.1990 | | M 022066 | | 1075 | | Monte Baldo | | 4 | | 12 | |
| I, Prov. Verona, Monte Baldo | 45.68806 N | 10.81361 E | H. Wittmann s.n., 11.07.2007 | | LI 623098 | | 1080 | | Monte Baldo | | 6 | | 16 | |
| I, Prov. Verona, Monte Baldo | 45.72639 N | 10.84389 E | G. van Buggenhout s.n., 07.1990 | | RO | | 1121 | | 0 | | #NV | | #NV | |
| I, Prov. Verona, Monte Baldo | 45.73379 N | 10.83423 E | G. van Buggenhout s.n., 07.1990 | | FI | | 1093 | | Monte Baldo | | 3 | | 11 | |
| I, Trient, Monte Bondone | 45.98806 N | 11.03083 E | H. Mittendorfer s.n., 17.06.1989 | | LI 036461 | | 1088 | | Monte Baldo | | 3 | | 11 | |
| I, Trient, Monte Tombea | 45.80611 N | 10.62722 E | H. Melzer s.n., 14.07.1955 | | LI 780784 | | 1085 | | Monte Baldo | | 7 | | 19 | |
| SLO, Kladivo | 46.43722 N | 14.34389 E | S. Novak s.n., 06.08.2009 | | LJM | | 1067 | | Slovenia | | 1 | | 1 | |
| SLO, Kofce gora | 46.41667 N | 14.41667 E | S. Novak s.n., 12.07.2009 | | LJM | | 1066 | | Slovenia | | 2 | | 8 | |
| SLO, Kofce gora | 46.41667 N | 14.41667 E | S. Novak s.n., 05.07.2009 | | LJM | | 1069 | | Slovenia | | 1 | | 1 | |
| SLO, Mojstrovka | 46.43944 N | 13.72111 E | S. Gebert s.n., 16.07.1983 | | HAL | | 1073 | | Slovenia | | #NV | | 4 | |
| SLO, Storzic | 46.35028 N | 14.40472 E | W. & H. Wittmann, R. & P. Resch s.n., 14.07.1994 | | LI 176259 | | 1087 | | Slovenia | | 7 | | 1 | |
| SLO, Tolste | 46.41667 N | 14.41667 E | S. Novak s.n., 08.07.2010 | | LJM | | 1068 | | Slovenia | | 1 | | 8 | |
|  |  |  |  | |  | |  | |  | |  | |  | |
| ***Helictotrichon sempervirens* (Villars) Pilger** | | | | |  | |  | |  | |  | |  | |
| F, Dépt. Drôme | 44.90000 N | 5.01667 E | M. Röser 2429, 22.08.1984 | | HAL | | 416 | | Drôme | | #NV | | 4 | |
| F, Dépt. Drôme, Lus-la-Croix-Haute | 44.67727 N | 5.73220 E | M. Röser 2421, 22.08.1984 | | HAL | | 1133 | | Drôme | | 7 | | 4 | |
| F, Dépt. Hautes Alpes, Col de Gleize | 44.62940 N | 6.04548 E | R. Karl s.n., 04.06.1995 | | GZU 212532 | | 1127 | | Hautes-Alpes | | 7 | | 4 | |
| F, Dépt. Hautes Alpes, Montagne d‘Aurose | 44.61745 N | 5.92918 E | H. Wittmann & A. Siebenbrunner s.n., 20.07.1983 | | LI 591166 | | 1144 | | Hautes-Alpes | | #NV | | 1 | |
| F, Dépt. Hautes-Alpes, Col Bayard | 44.61501 N | 6.07187 E | M. Röser 2186, 04.08.1984 | | HAL | | 1134 | | Hautes-Alpes | | 7 | | 4 | |
| F, Dépt. Hautes-Alpes, Combe d’ Aurouse | 44.60992 N | 5.93176 E | D. Aeschimann 4930, s.d. | | G 00428387 | | 1162 | | Hautes-Alpes | | 7 | | 4 | |
| F, Dépt. Hautes-Alpes, Pic de Bure | 44.60992 N | 5.93176 E | M. Röser 2206, 05.08.1984 | | HAL | | 1136 | | Hautes-Alpes | | 7 | | 4 | |
| F, Dépt. Hautes-Alpes, Piolit | 44.60338 N | 6.27173 E | D. Aeschimann 4799, s.d. | | G 00428386 | | 1163 | | Hautes-Alpes | | #NV | | 2 | |
| F, Dépt. Hautes-Alpes, Valley Réallon | 44.60186 N | 6.35816 E | M. Röser 2409, 21.08.1984 | | HAL | | 1158 | | Hautes-Alpes | | 7 | | 6 | |
| F, Maritime Alps, Baus de la Frema | 44.08366 N | 7.21901 E | H. Wittmann s.n., 26.07.2000 | | LI 563031 | | 1143 | | Maritime Alps | | 10 | | 2 | |
| F, Maritime Alps, Col de la Cayolle | 44.25916 N | 6.74371 E | M. Röser 2297, 13.08.1984 | | HAL | | 1157 | | Maritime Alps | | 7 | | 4 | |
| F, Maritime Alps, Col de Turini | 43.97717 N | 7.40783 E | M. Röser 6710, 21.07.1989 | | HAL | | 1132 | | Maritime Alps | | #NV | | 4 | |
| F, Maritime Alps, Col des Champs | 44.15950 N | 6.71891 E | M. Röser 2294, 13.08.1984 | | HAL | | 1135 | | Maritime Alps | | 7 | | 4 | |
| F, Mont Tournairet, Tête de Clans | 44.04083 N | 7.20778 E | G. Pils 1156, 09.07.1996 | | KL 119414 | | 1149 | | Hautes-Alpes | | 7 | | 2 | |
|  |  |  |  | |  | |  | |  | |  | |  | |
| ***Helictotrichon setaceum* (Villars) Henrard subsp. *petzense* (Melzer) Röser** | | | | |  | |  | |  | |  | |  | |
| A, Carinthia, Hochstuhl | 46.44389 N | 14.18806 E | L. Kutschera s.n., 30.06.1994 | | KL 175651 | | 1147 | | Karawanks | | 7 | | 1 | |
| A, Carinthia, Kärntner Storschitz | 46.43111 N | 14.52528 E | P. Gutte & W. Morawetz 124/97, 08.07.1997 | | LZ 157917 (specimen A) | | 1125 | | Karawanks | | 10 | | 23 | |
| A, Carinthia, Kärntner Storschitz | 46.43111 N | 14.52528 E | P. Gutte & W. Morawetz 124/97, 08.07.1997 | | LZ 157917 (specimen B) | | 1126 | | Karawanks | | 10 | | 24 | |
| A, Carinthia, Kärntner Storschitz | 46.42861 N | 14.52361 E | B. Heuchert 1303, 04.08.2001 | | HAL | | 1131 | | Karawanks | | 10 | | 24 | |
| A, Carinthia, Kärntner Storschitz | 46.42861 N | 14.52361 E | B. Heuchert 1302, 04.08.2001 | | HAL | | 81 | | Karawanks | | #NV | | 4 | |
| A, Karawanks, Obere Krischa | 46.50972 N | 14.76111 E | H. Wagner s.n., Sommer 1987 | | LI 010862 | | 1122 | | Karawanks | | 10 | | 22 | |
| A, Karawanks, Petzen | 46.51056 N | 14.77028 E | B. Heuchert 803, 03.08.2001 | | HAL | | 1129 | | Karawanks | | 10 | | 25 | |
| A, Karawanks, Petzen | 46.51000 N | 14.77222 E | B. Heuchert 1003, 03.08.2001 | | HAL | | 1130 | | Karawanks | | 10 | | 26 | |
| A, Karawanks, Petzen | 46.50972 N | 14.77000 E | M. Röser 10646, 09.07.1998 | | HAL | | 199 | | Karawanks | | #NV | | 4 | |
| A, Karawanks, Uschowatore | 46.45083 N | 14.67111 E | H. Melzer s.n., 27.08.1990 | | LI | | 1139 | | Karawanks | | 10 | | 27 | |
|  |  |  |  | |  | |  | |  | |  | |  | |
| ***Helictotrichon setaceum* (Villars) Henrard subsp. *setaceum*** | | | | |  | |  | |  | |  | |  | |
| F, Dépt. Drôme, Col de Rousset | 44.83860 N | 5.40603 E | M. Röser 2261, 10.08.1984 | | HAL | | 831 | | Drôme | | #NV | | 4 | |
| F, Dépt. Drôme, Lus-la-Croix-Haute | 44.67660 N | 5.73813 E | M. Röser 2420, 22.08.1984 | | HAL | | 567 | | Drôme | | 8 | | 4 | |
| F, Dépt. Vaucluse, Mont Ventoux | 44.17397 N | 5.27828 E | M. Röser 10631, 20.08.1997 | | HAL | | 830 | | Vaucluse | | 8 | | 4 | |
| F, Dépt. Vaucluse, Mont Ventoux | 44.17397 N | 5.27828 E | M. Nydegger 30648, 12.07.1986 | | M 0222071 | | 1123 | | Vaucluse | | 10 | | 4 | |
| F, Dépt. Vaucluse, Mont Ventoux | 44.17397 N | 5.27828 E | M. Nydegger 30648, s.d. | | G 00428385 | | 1161 | | Vaucluse | | #NV | | 4 | |
| F, Maritime Alps, Col de Tende | 44.13777 N | 7.56874 E | K. Lewejohann SOF-73-273, 27.07.1973 | | LI 548386 | | 1156 | | Maritime Alps | | #NV | | 28 | |
| F, Maritime Alps, Mont Chajol | 44.10292 N | 7.52884 E | M. Staudinger s.n., 08.07.1998 | | LI 484940 | | 1141 | | Maritime Alps | | 10 | | 4 | |
| F, Maritime Alps, Monte Chajol | 44.09981 N | 7.53061 E | W. Guttermann 32606 & G. Schneeweiß, P. Schönswetter, A. Tribsch , 08.07.1998 | | WU 082320 | | 1128 | | Maritime Alps | | 7 | | 4 | |
| F, Maritime Alps, Mt. Agnelet | 44.08425 N | 7.533628 E | F. Krendl s.n., 20.07.1968 | | LI 329648 | | 1155 | | Maritime Alps | | 7 | | 4 | |
| F, Mont Picogu | 43.86511 N | 6.6318 E | G. Pils 1197, 12.07.1996 | | KL 119089 | | 1148 | | Hautes-Alpes | | #NV | | 4 | |
| I, Piedmont, La Motta | 44.20000 N | 7.56667 E | F. Krendl s.n., 01.07.1982 | | LI 410757 | | 1142 | | NW Italy | | 7 | | 2 | |
