# Supplementary material for: Glacial refugia and speciation in a group of wind-pollinated and -dispersed, endemic Alpine species of Helictotrichon (Poaceae)
Source: PLoS One. 2018 Oct 15;13(10):e0205354. doi: 10.1371/journal.pone.0205354 (PMC6188759; doi:10.1371/journal.pone.0205354)
Supplement: S3 Table — NE, Northeast; NW, Northwest. (DOCX) [file pone.0205354.s003.docx]

**Supporting Information**

**S3 Table. Sampled populations for the phylogeographic analyses of the Alpine *Helictotrichon* species.** NE, Northeast; NW, Northwest.

| ID | Country | Population | Latitude | Longitude |
| --- | --- | --- | --- | --- |
| 1 | France | Drome | 44.9000 | 5.0167 |
| 2 | France | Vaucluse | 44.1740 | 5.2783 |
| 3 | France | Savoie | 44.6410 | 6.2077 |
| 4 | Switzerland | Maritime Alps | 45.1635 | 6.2677 |
| 5 | France | Hautes-Alpes | 44.8225 | 6.7872 |
| 6 | Italy | NW Italy | 45.0647 | 7.1067 |
| 7 | Italy | Lombardy | 45.9536 | 9.5122 |
| 8 | Germany | Bavaria | 47.4200 | 10.3675 |
| 9 | Italy | Monte Baldo | 45.7338 | 10.8342 |
| 10 | Italy | NE Italy | 46.4588 | 11.8755 |
| 11 | Austria | Tyrol | 47.6097 | 12.5086 |
| 12 | Austria | Salzburg | 47.4669 | 13.3547 |
| 13 | Austria | Salzkammergut | 47.7768 | 13.4307 |
| 14 | Austria | Upper Austria | 47.8858 | 14.2569 |
| 15 | Austria | Eisenerzer Alpen | 47.6900 | 15.6900 |
| 16 | Austria | Schneeberg | 47.7539 | 16.2375 |
| 17 | Slovenia | Slovenia | 46.4372 | 13.9439 |
| 18 | Austria | Karawanks | 46.4469 | 14.4294 |
| 19 | Austria | Kaerntner Storschitz | 46.4311 | 14.5253 |
