# Supplementary material for: Glacial refugia and speciation in a group of wind-pollinated and -dispersed, endemic Alpine species of Helictotrichon (Poaceae)
Source: PLoS One. 2018 Oct 15;13(10):e0205354. doi: 10.1371/journal.pone.0205354 (PMC6188759; doi:10.1371/journal.pone.0205354)
Supplement: S2 Fig — Kinship coefficients between individuals are standardized relative to the unweighted average allele frequencies over species. (DOCX) [file pone.0205354.s005.docx]

**Supporting Information**

**S2 Figure.** **Average kinship N(d) between individuals of *Helictotrichon parlatorei* according to geographical distances based on combined chloroplast sequences (*rps16, rpL*32-*trnL* (UAG), *ycf*3ln1, *ycf*3ln2).** Kinship coefficients between individuals are standardized relative to the unweighted average allele frequencies over species.
